# Intricacies of the Molecular Machinery of Catecholamine Biosynthesis and Secretion by Chromaffin Cells of the Normal Adrenal Medulla and in Pheochromocytoma and Paraganglioma

**DOI:** 10.3390/cancers11081121

**Published:** 2019-08-06

**Authors:** Annika M.A. Berends, Graeme Eisenhofer, Lauren Fishbein, Anouk N.A. van der Horst-Schrivers, Ido P. Kema, Thera P. Links, Jacques W.M. Lenders, Michiel N. Kerstens

**Affiliations:** 1 Department of Endocrinology, University of Groningen, University Medical Center Groningen, 9700 RB Groningen, The Netherlands; 2 Department of Clinical Chemistry and Laboratory Medicine and Department of Medicine III, University Hospital Carl Gustav Carus, Technical University Dresden, 01069 Dresden, Germany; 3 Division of Endocrinology, Metabolism and Diabetes and Division of Biomedical Informatics and Personalized Medicine, Department of Medicine, University of Colorado School of Medicine, University of Colorado Cancer Center, Aurora, CO 80045, USA; 4 Department of Laboratory Medicine, University of Groningen, University Medical Center Groningen, 9700 RB Groningen, The Netherlands; 5 Department of Medicine III, University Hospital Carl Gustav Carus, Technical University Dresden, 01307 Dresden, Germany; 6 Department of Internal Medicine, Radboud University Medical Center, 6525 GA Nijmegen, The Netherlands

**Keywords:** PPGL, catecholamines, adrenomedullary function

## Abstract

The adrenal medulla is composed predominantly of chromaffin cells producing and secreting the catecholamines dopamine, norepinephrine, and epinephrine. Catecholamine biosynthesis and secretion is a complex and tightly controlled physiologic process. The pathways involved have been extensively studied, and various elements of the underlying molecular machinery have been identified. In this review, we provide a detailed description of the route from stimulus to secretion of catecholamines by the normal adrenal chromaffin cell compared to chromaffin tumor cells in pheochromocytomas. Pheochromocytomas are adrenomedullary tumors that are characterized by uncontrolled synthesis and secretion of catecholamines. This uncontrolled secretion can be partly explained by perturbations of the molecular catecholamine secretory machinery in pheochromocytoma cells. Chromaffin cell tumors also include sympathetic paragangliomas originating in sympathetic ganglia. Pheochromocytomas and paragangliomas are usually locally confined tumors, but about 15% do metastasize to distant locations. Histopathological examination currently poorly predicts future biologic behavior, thus long term postoperative follow-up is required. Therefore, there is an unmet need for prognostic biomarkers. Clearer understanding of the cellular mechanisms involved in the secretory characteristics of pheochromocytomas and sympathetic paragangliomas may offer one approach for the discovery of novel prognostic biomarkers for improved therapeutic targeting and monitoring of treatment or disease progression.

## 1. Introduction

The adrenal medulla occupies the central portion of the adrenal gland and accounts for about 10% of total adrenal gland volume [1]. The adrenal medulla is essentially a specialized sympathetic ganglion releasing hormones in response to neural input and therefore is an integral part of the autonomic nervous system [2,3]. The adrenomedullary chromaffin cells are embryologically derived from migrating neural crest cells that develop into sympathoadrenal progenitors [4,5]. These sympathoadrenal progenitor cells also give rise to the chromaffin cells present in the sympathetic chain and prevertebral paraganglia. During adrenal organogenesis, close interactions between its two components, medulla and cortex, are necessary for differentiation, morphogenesis, and survival of the adrenal gland. This cortical–chromaffin crosstalk remains important for physiological regulation of adrenal hormone biosynthesis in adult life and also is relevant for the pathogenesis of various adrenal gland disorders [6,7,8,9]. One of the histological infrastructural requirements for this crosstalk is the centripetally directed arterial blood flow from adrenal cortex to medulla. In addition, cortical cells are diffusely present in the adrenal medulla and, conversely, chromaffin cells are intermixed with cortical cells within all three zones of the adrenal cortex [10].

The principal function of the adrenal medulla is the biosynthesis and the secretion into the circulation of the catecholamine epinephrine [6,11]. Epinephrine has a crucial role in the “fight-or-flight” response, which allows an organism to adapt to stressful conditions. The acute rise in epinephrine in response to physical or psychological stress stimuli results in hemodynamic and metabolic effects that modulate various functions such as blood pressure, cardiac output, and blood glucose by acting on cells expressing α- and β- adrenergic receptors [12,13]. Under basal conditions, however, epinephrine functions as a circulating metabolic hormone, and it is the norepinephrine secreted by sympathetic nerves acting immediately in the vicinity of exocytotic secretion that is the catecholamine mainly regulating cardiovascular function. The norepinephrine that escapes re-uptake processes to enter the circulation has negligible impact on the cardiovascular system. Nevertheless, both norepinephrine and epinephrine secreted by pheochromocytomas in excessive amounts directly into the circulation can have profound effects on cardiovascular function, with further impacts of co-secreted peptides. From a clinical perspective, these tumors are the most important disease of the adrenal medulla. Chromaffin cell tumors may also arise in extra-adrenal sympathetic paraganglia, in which case they are termed sympathetic paragangliomas [14,15,16].

A cardinal feature of chromaffin cell tumors is their capacity to produce and secrete excessive amounts of catecholamines, which may evoke signs and symptoms such as paroxysmal hypertension, sweating, and tachycardia. The hypersecretion of catecholamines may cause acute, life-threatening blood pressure elevations and arrhythmias and is associated with a significantly increased rate of cardiovascular morbidity and mortality [17,18,19,20].

Pheochromocytomas and sympathetic paragangliomas are rare neuroendocrine tumors with respective reported annual incidences of 0.46 and 0.11 per 100,000 individuals [21]. Detected incidence of pheochromocytomas has doubled during the past two decades, most likely a result of changes in diagnostic practices leading to earlier detection. The cornerstone of biochemical diagnosis of a pheochromocytoma or a sympathetic paraganglioma is the demonstration of elevated plasma or urinary concentrations of metanephrine, normetanephrine, or 3-methoxytyramine, i.e., the O-methylated metabolites of epinephrine, norepinephrine, or dopamine, respectively [22]. Several anatomical and functional imaging studies are available for localization of the tumor, after which curative treatment by surgical resection can be offered [23].

Pheochromocytomas and paragangliomas are highly heterogeneous neuro-endocrine tumors with regards to possible anatomic location, genetic context, symptomatology, metastatic potential, and the degree of catecholamine release. Genetic mutations play a critical role in tumorigenesis and affect various metabolic pathways, which also result in different mutation-dependent biochemical phenotypes [3,24,25,26].

In recent years, our knowledge of the genotype–phenotype interrelationship and metabolomics of these intriguing neuro-endocrine tumors has expanded rapidly. Nevertheless, there are still several areas of uncertainty. For instance, in the absence of metastases, it is difficult to predict whether a pheochromocytoma or a paraganglioma will demonstrate a benign or a malignant clinical course [16]. There are no clear-cut pathological markers to establish malignancy with certainty at first presentation. Also, there is no straightforward relationship between the biochemical phenotype of a pheochromocytoma or sympathetic paraganglioma and the associated signs or symptoms [3]. In the present review, we aim to provide a detailed picture of the pathways involved in catecholamine production and secretion in normal adrenomedullary chromaffin cells. We also visit what is known about the molecular perturbations in catecholamine biosynthesis and secretion in pheochromocytoma and sympathetic paraganglioma. Improved understanding of these mechanisms at the molecular level might provide insight into associated pathological complications, clarify highly variable presentations, and aid in identification of new diagnostic or therapeutic strategies for personalized care.

## 2. Adrenomedullary Function

### 2.1. Biosynthesis of Catecholamines

**Figure 1:**
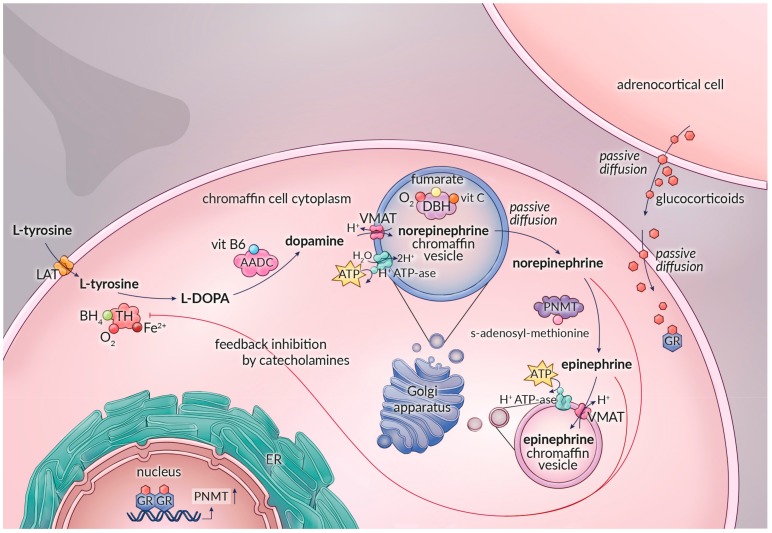
The catecholamine biosynthetic pathway in an adrenomedullary chromaffin cell or a pheochromocytoma cell. Norepinephrine and epinephrine are stored in separate chromaffin storage vesicles. Abbreviations: LAT: L-type amino acid transporter; TH: tyrosine hydroxylase; L-DOPA: L-3,4-dihydroxyphenylalanine; AADC: aromatic L-amino acid decarboxylase; DBH: dopamine β-hydroxylase; PNMT: phenylethanolamine-N-methyltransferase; BH4: tetrahydrobiopterin; 02: molecular oxygen; VitB6: pyridoxalphosphate; VitC: ascorbate; VMAT: vesicular monoamine transporters; GR: glucocorticoid receptor.

Adrenomedullary catecholamine biosynthesis starts with uptake of the nonessential amino acid L-tyrosine by the chromaffin cell. L-tyrosine is obtained from food sources or is derived from the essential amino acid phenylalanine through the activity of phenylalanine hydroxylase, which is mainly expressed in liver, kidney, and pancreas [27,28]. L-tyrosine is transported into the cytoplasm of the adrenal chromaffin cell by the membrane bound L-type amino acid transporter system (LAT1 and LAT2) [29,30]. Catecholamine biosynthesis involves the sequential activity of four enzymes: tyrosine hydroxylase (TH), aromatic L-amino acid decarboxylase (AADC), dopamine β-hydroxylase (DBH), and phenylethanolamine-*N*-methyltransferase (PNMT). Except for DBH, all these enzymes are localized in the cytoplasm of the chromaffin cell (Figure 1). The end products of this biosynthetic route are dopamine, norepinephrine, or epinephrine, depending on intracellular enzyme expression. Epinephrine is mainly produced by the adrenomedullary chromaffin cells (>95%) and functions as a hormone released directly into the bloodstream. In contrast, circulating norepinephrine is mainly derived from overflow of the neurotransmitter from sympathetic nerve endings with adrenomedullary chromaffin cell production providing usually a less than 10% contribution [11,13,31].

#### 2.1.1. Tyrosine Hydroxylase

The initial and rate limiting step in catecholamine biosynthesis is the conversion of L-tyrosine to L-3,4-dihydroxyphenylalanine (L-DOPA) by tyrosine hydroxylase (TH, EC 1.14.16.2, molecular mass of approximately 240 kDa) [32,33]. Locations of catecholamine biosynthesis are therefore dependent on the expression of TH, which is largely confined to postganglionic sympathetic nerve endings and adrenal and extra-adrenal chromaffin cells. In the adrenal medulla, this enzyme has a Km of 2 × 10^−5^ mol/L [27]. For this specific hydroxylation step, TH requires tetrahydrobiopterin, molecular oxygen, and Fe^2+^ as cofactors. Tetrahydrobiopterin is synthesized from guanosine triphosphate (GTP) and serves as a donor for hydrogen atoms to maintain TH in a reduced and active state [27,34]. The human *TH* gene is located at chromosome 11p15.5 and contains 13 exons [35], with four isoforms produced by alternative mRNA splicing.

Regulation of TH activity is an important way to control catecholamine biosynthesis. This is a complex process encompassing multiple modes of regulation. Short-term post-transcriptional mechanisms include feedback inhibition by catecholamines, enzyme phosphorylation and dephosphorylation, as well as ubiquitination. Long-term regulation mainly involves transcriptional mechanisms [36]. The ubiquitin–proteasome pathway is thought to be involved in the degradation of TH [37]. Catecholamines exert negative feedback control through oxidation of tetrahydrobiopterin to pteridine, thereby preventing the formation of TH in its reduced active state [27]. In addition, catecholamines act as competitive antagonists of tetrahydrobiopterin at the active site of the catalytic domain of TH [27]. Short-term regulation of TH activity is also achieved by a mechanism of phosphorylation and dephosphorylation of one or more of the four serine residues at the regulatory site of TH. Phosphorylation is catalyzed by multiple kinases (e.g., PKA, PKC, CaMKII, MAPKAP-K2, ERK1, ERK2, MSK1, PRAK) and results in release from the feedback inhibition by catecholamines, thereby stimulating enzyme activity.

Dephosphorylation by phosphatase PP2A, and to a lesser extent by PP2C, restores catecholaminergic inhibition of the TH enzyme [36]. This negative feedback is mediated via alpha2-adrenergic or D2-dopaminergic receptors, which activate cyclic adenosine monophosphate (cAMP) or Ca^2+^/calmodulin-dependent protein phosphatases [27]. Prolonged stimulation of catecholamine biosynthesis results in induction of TH protein synthesis through several cAMP dependent pathways activating *TH* gene transcription [27,34,36,38,39,40].

Given the importance of its activity to catecholamine synthesis and the complexity of its regulation, TH has gained great interest in many fields of biomedical research. Recent studies, for example, have demonstrated the presence of several TH polymorphisms in the general population, some of which appear associated with increased norepinephrine levels and elevated blood pressure [35,41].

#### 2.1.2. Aromatic L-Amino Acid Decarboxylase

The next step in catecholamine biosynthesis is the decarboxylation of L-DOPA to dopamine by cytosolic aromatic L-amino acid decarboxylase (AADC; EC 4.1.1.28). For this conversion, pyridoxalphosphate (vitamin B6) is required as a cofactor [27,42]. AADC is a 100 kDa homodimeric protein encoded by a single gene located at chromosome 7p12.1 with a Km of 4 × 10^−4^ mol/L [42,43]. The calculated Km greatly exceeds the endogenous concentration of L-DOPA, which means that the AADC enzyme is not fully saturated, and the rate at which dopamine can be synthesized is therefore limited by the availability of L-DOPA as a substrate [42]. The AADC enzyme has a wide tissue distribution and is not specific for chromaffin cells [42,44].

Short-term regulation of AADC enzyme activity by a previously postulated mechanism involving cAMP or phosphorylation by protein kinases seems to play no significant role in the regulation of the catecholamine biosynthetic pathway in situ [42,45]. The added value of AADC enzyme upregulation by modulation of gene expression for physiological demands to increase catecholamine production in postganglionic sympathetic nerve endings remains questionable [45,46,47].

#### 2.1.3. Dopamine β-hydroxylase

In adrenal chromaffin cells, dopamine is further catalyzed to norepinephrine by dopamine β-hydroxylase (DBH; EC 1.14.17.1). Because of the intravesicular location of DBH, dopamine first must be translocated into norepinephrine storage vesicles by vesicular monoamine transporters (VMATs) [11]. The intravesicular conversion of dopamine represents the final step in the biosynthesis of norepinephrine. DBH, a mixed-function oxidase, is a 290 kDa copper protein with a Km of 8.4 × 10^−4^ mol/L and utilizes molecular oxygen, fumarate, and L-ascorbic acid as its main cofactors [27,48,49]. These requirements for L-ascorbic acid and fumarate are not specific. Catechol (i.e., pyrocatechol or 1,2 dihydroxybenzene) seems to be a weak substitute for L-ascorbic acid and other activating anions, such as acetate and chloride, which can replicate the effects of fumarate at least partially [27,48,50,51,52].

In humans, DBH is encoded by a gene located at chromosome 9q34.2. Increased catecholamine biosynthesis in response to stress is associated with increased levels of mRNAs encoding catecholamine synthesizing enzymes. In adrenomedullary chromaffin cells, this response to stress is rapid, especially for TH and PNMT [39]. Previous studies also revealed upregulation of adrenal *DBH* gene expression by various transcriptional mechanisms in response to prolonged or repeated stressors [39,53]. However, in contrast to *TH*, short or intermediate duration of stress does not result in a significant increase of *DBH* mRNA [39].

#### 2.1.4. Phenylethanolamine-*N*-Methyltransferase

Norepinephrine formed in the chromaffin vesicles diffuses passively into the cytosol, where it is converted to epinephrine by the enzyme phenylethanolamine-*N*-methyl transferase (PNMT; EC 2.1.1.28) [11,24]. PNMT, which has a predicted molecular weight of 30.9 kDa and a Km of 9.2 × 10^−6^ mol/L, requires *S*-adenosylmethionine as a methyl donor and cosubstrate [27,54,55]. PNMT is not substrate specific and also is involved in the biosynthesis of other *N*-methylated trace amines [11,27]. Expression of PNMT is controlled by glucocorticoid receptor-mediated mechanisms, acting in concert with several other transcription factors such as Egr-1, AP2, Sp1, and MAZ [6,39,56,57]. The proximity of adrenocortical cells to the adrenal medulla guarantees high circulating glucocorticoid levels, which cross the chromaffin cell membrane through passive diffusion. Glucocorticoid binds to the intracytoplasmatic glucocorticoid receptor, and the receptor–hormone complex migrates to the cell nucleus and binds to the glucocorticoid response element of the promoter region of the *PNMT* gene located on chromosome 17q12, activating gene transcription [7,58,59]. This explains why the adrenal gland is the body’s most important source of epinephrine, whereas the expression of extra-adrenal PNMT is limited to a small number of neurons in the central nervous system and to a subset of cardiomyocytes [6,60,61].

#### 2.1.5. Co-Secreted Products

Chromaffin cells of the adrenal medulla synthesize a large variety of other substances, such as neurotransmitters, enzymes, peptides, and proteins, which are also stored in chromaffin vesicles and co-secreted along with catecholamines [62,63,64]. Over the past decades, the components of this vesicular cocktail have been studied in great detail (Table 1).

Among these substances, chromogranin A and the trophic and secretion stimulating peptides pituitary adenylate cyclase-activating polypeptide (PACAP), neuropeptide Y (NPY), and adrenomedullin (AM) have gained the most attention because of their endocrine, paracrine, and autocrine effects, their importance for vesiculogenesis, and their possible roles in neoplastic chromaffin cell proliferation, differentiation, and survival, as further discussed below [62,63,64,65,101,113,129,157].

### 2.2. Storage and Secretion of Catecholamines

#### 2.2.1. Storage and Vesicular Transmembrane Dynamics

In adrenomedullary chromaffin cells, catecholamines are stored in specialized vesicles. The bidirectional vesicular–cytosolic exchange of catecholamines is a dynamic process of active uptake into these chromaffin storage vesicles and passive leakage from vesicles into the cytosol [3]. After synthesis, dopamine and epinephrine are actively transported from the cytosol into chromaffin storage vesicles by vesicular monoamine transporters (VMAT1 and VMAT2) [158,159]. The driving force for this active transport is provided by an ATP-dependent vesicular membrane proton pump that maintains a transvesicular hydrogen ion (H^+^) electrochemical gradient by acidifying the vesicle matrix. Vesicular uptake for catecholamines via VMAT is accompanied by exchange of an H+ ion from the vesical matrix towards the cytosol [62,160] (Figure 1).

#### 2.2.2. Characteristics of Chromaffin Storage Vesicles

Chromaffin storage vesicles are highly specialized organelles of the chromaffin cells for storage and exocytosis. These membrane-bound electron-dense organelles originate from the Golgi network and are 150 to 350 nm in diameter. Each adrenomedullary chromaffin cell contains about 12,000 to 30,000 of these vesicles, corresponding (on average) to 13.5% of the cytoplasmic cell volume [63,65].

The adrenal medulla of some species harbors two distinct populations of chromaffin cells, which either produce epinephrine or norepinephrine depending on the presence or the absence of PNMT [3,11,63]. The proportion of epinephrine versus norepinephrine producing chromaffin cells in the adrenal medulla varies between species, but the adrenergic phenotype usually predominates [3,6,62,63,161]. This is particularly so in humans, where most chromaffin cells appear to have mixed function [6,11,62,161,162]. Nevertheless, there are clear ultrastructural differences between epinephrine and norepinephrine-containing vesicles when studied by electron microscopy. Epinephrine-containing vesicles are round or elongated in shape and demonstrate fine granular, medium-density vesicles with a characteristic narrow and uniform peripheral halo, whereas norepinephrine-containing vesicles demonstrate a high density and homogeneous content with a limiting membrane, which may be separated from the matrix constituents by a prominent lucent halo.

The mechanisms initiating and regulating the biogenesis of chromaffin vesicles are largely unknown. It is believed that structural proteins of the granin family, in particular chromogranin A, have an important role in vesiculogenesis, providing structural domains that drive chromaffin vesicle formation in the Golgi network. Furthermore, the ability of chromogranin A to bind catecholamines is thought to regulate stability of the vesicle by reducing osmotic pressure, thereby preventing vesicles from bursting; it is also thought to protect catecholamines against enzymatic degradation until secretion is warranted [27,63,65].

After the formation of chromaffin vesicles, maturation continues with catecholamine synthesis and storage not occurring until late in vesicle formation [63]. Fully matured chromaffin vesicles remain in the chromaffin cell until stimulation for exocytosis [63,65].

The molecular composition of chromaffin vesicles is complex. Besides catecholamines, intravesicular contents include a diverse mixture of peptides, proteases, enzymes, and granins (chromogranins, secretogranins) with a multiplicity of functions (Table 1). The high concentration of regulatory and modulating peptides and proteins reflects broad endocrine, paracrine, and autocrine functions of adrenomedullary chromaffin vesicles. The physiological processes modulated by these constituents not only involve the fine-tuning of catecholamine biosynthesis and secretion but also encompass various analgesic, immunomodulatory, antimicrobial, and anti-inflammatory responses to cell stress [62,63].

#### 2.2.3. Secretion and Re-Uptake of Catecholamines

After exocytosis of storage vesicles and secretion of catecholamines into the bloodstream, norepinephrine and epinephrine are removed from the circulation by neural and extra-neuronal monoamine transporters and are inactivated by metabolizing enzymes [163]. The re-uptake mechanism through the norepinephrine transporter (NET) by catecholamine synthesizing cells is only relevant in sympathetic postganglionic and central nervous system neurons and provides rapid termination of the neurotransmitter signal at the postsynaptic membrane and enables recycling of catecholamines for re-release. NET is not only located presynaptically but also at several extraneuronal sites, including the adrenal medulla [31,163,164]. The precise function of the NET in the adrenal gland is, however, not entirely clear. A detailed discussion of reuptake as well as pre- and postsynaptic effects of catecholamines is beyond the scope of the current review, and for further reading, we refer to the literature [163,165,166].

### 2.3. Regulation of Adrenomedullary Activity

#### 2.3.1. Stimulus-Dependent Exocytosis in Adrenal Chromaffin Cells

Exocytosis of chromaffin storage vesicles is a tightly controlled process. Under basal conditions, only a few secretory vesicles are released into the circulation, resulting in a catecholamine secretion rate in the order of nanograms per minute [117]. Toxic effects of excessive chronic catecholamine release such as in heart failure, pulmonary edema, and malignant hypertension have been reported from incessant circulating catecholamine concentrations of 10^−6^ mol/L or more, corresponding to the release of 5% of all adrenomedullary chromaffin vesicles [167]. In acute stress situations, the amount of released vesicles from the total adrenal gland can be temporarily greatly increased, with plasma catecholamine concentrations reaching up to 60 times more than normal [117,167]. Well-known stimuli that activate the exocytotic process are hypoglycaemia, hypovolemia, hypotension, hypoxemia, and severe pain or emotional distress [3].

The rather complex mechanisms regulating chromaffin cell exocytotic machinery are executed at neuronal and non-neuronal levels [145,167,168,169]. It is thought that each adrenomedullary chromaffin cell receives its own individual neuronal and non-neuronal input [167].

**Figure 2:**
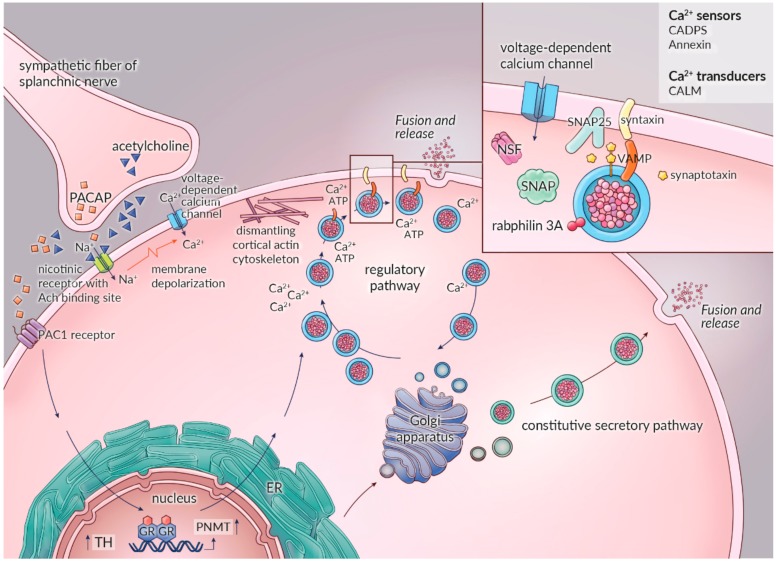
Schematic overview of the stimulation–secretion coupling in the adrenomedullary chromaffin cell with the multiple functionally definable stages and the different secretory pathways. Abbreviations: ER: endoplasmic reticulum; Ach: acetylcholine; VAMP: vesicle-associated membrane protein; SNAP: synaptosomal-associated protein; NSF: N-ethylmaleimide Soluble Factor proteins; CADPS: Ca^2+^ dependent secretion activator; CALM: calmodulin; PACAP: pituitary adenylate cyclase-activating polypeptide; PAC1 receptor: PACAP-preferring receptor; GR: glucocorticoid receptor.

At a neuronal level, adrenomedullary chromaffin cells are innervated by the cholinergic preganglionic sympathetic fibers of the splanchnic nerve. One single chromaffin cell can receive input by up to five synapses [170]. Acetylcholine released by these nerve endings predominantly binds to nicotinic receptors on the chromaffin cell, resulting in membrane depolarization with subsequent calcium influx followed by stimulation of exocytosis and catecholamine secretion. Cholinergic receptors of the muscarinic type are also expressed on the chromaffin cell, but their contribution to catecholamine secretion is less important. Further enhancement of the stimulation–secretion coupling is provided by propagation of the secretion signal via gap junctions between chromaffin cells formed by connexins, which are specific proteins involved in cell-to-cell communication. This intercellular communication can be upregulated in stressful conditions [170]. Besides acetylcholine, splanchnic nerve terminals also contain PACAP as a neurotransmitter. This neuropeptide is not only stored in chromaffin vesicles and co-secreted with catecholamines but also acts as an important neurotransmitter at the splanchnic medullary synapse, where it activates the PACAP-preferring receptor (PAC1-R) on the postsynaptic membrane of the chromaffin cell. Laboratory experiments have shown that PACAP is only released at high frequencies of nerve stimulation, which is the firing rate occurring in stress conditions. In contrast to the acetylcholine evoked catecholamine secretion, adrenomedullary stimulation by PACAP is not susceptible to desensitization, which ensures robust catecholamine release under conditions of continuous stress (Figure 2) [40,130].

Non-neuronal regulation of exocytosis occurs predominantly through autocrine or paracrine routes. As a result, cellular catecholamine secretion is partially under the influence of the exocytotic activity of neighboring chromaffin cells [167]. Furthermore, both lipopolysaccharide and cytokine receptors were recently demonstrated on chromaffin cells, pointing towards a role of the adrenal medulla in the complex regulation of the inflammatory stress response [40].

In general, the exocytotic secretion of vesicular contents of adrenomedullary chromaffin cells can be achieved via regulated and constitutive secretory pathways. The regulated secretory pathway provides the principle mechanism responsible for controlled release of catecholamines and is calcium-dependent and responsive to both neuronal and non-neuronal input. In contrast, the constitutive secretory pathway, which is calcium-independent, is mainly unresponsive to neuronal and non-neuronal input. The principal function of the constitutive secretory pathway is thought to be the transport of proteins and macromolecules to the cell surface for purposes of membrane maintenance and support of the extracellular matrix. In addition, this pathway may also contribute to basal release of catecholamines (Figure 2) [145,169].

#### 2.3.2. Neuronal Regulation of the Calcium-Dependent Catecholamine Secretory Pathway

In recent years, considerable progress has been made in unravelling the complex molecular background and the functional elements of the highly regulated exocytotic machinery in adrenomedullary chromaffin cells (Figure 2) [145,171,172]. Release of acetylcholine by the splanchnic nerve activates the nicotinic receptor on chromaffin cells, resulting in opening of the ionophoric part of the receptor protein, thereby allowing the entry of extracellular sodium (Na^+^) and calcium (Ca^2+^). This generates a small membrane depolarization, resulting in the opening of voltage dependent Na^+^ channels. The subsequent Na^+^ influx results in a large membrane depolarization, which opens various types of voltage dependent Ca^2+^ channels [167].

The distribution of the different calcium channel subtypes is species specific. The P/Q-type calcium channel predominates in human adrenomedullary chromaffin cells [167,171,172]. As a consequence of elevated intracellular Ca^2+^ concentrations, the exocytotic machinery and the regulatory components for vesicular exocytosis are activated, which occurs through a pathway consisting of secretory vesicle recruitment, docking, priming, and fusion with the plasma membrane. Priming is the process in which secretory vesicles become fusion competent [167,172]. First, Ca^2+^ influx leads to dismantling of the cortical actin cytoskeleton of the chromaffin cell, a dynamic network of numerous cytoplasmic proteins located on the inner face of the chromaffin cell membrane [173]. Although this can be activated without Ca^2+^, this is mainly an ATP and a Ca^2+^ dependent step, as are recruitment, tethering, and docking of the vesicles.

Fusion, release, and retrieval of vesicles can be triggered by Ca^2+^ in the absence of ATP [145,174]. Various soluble and membrane-bound proteins are involved in the complex protein–protein interactions underlying membrane trafficking and fusion. Key components of this process are *N*-ethylmaleimide soluble factor proteins (soluble cytosolic NSF), soluble NSF attachment proteins (SNAPs), and soluble NSF attachment receptor proteins (SNAREs). The vesicle-associated membrane protein (VAMP or synaptobrevin) and the calcium binding protein synaptotagmin are SNAREs located at the secretory vesicle membrane. The synaptosomal-associated protein 25 (SNAP-25) and syntaxin are SNAREs acting on the chromaffin cell plasma membrane [145,167,172]. The complex formed by chromaffin vesicle SNAREs (VAMP, synaptotagmin), the chromaffin cell membrane SNAREs (syntaxin, SNAP-25), and the cytosolic proteins (NSF) is thought to provide the primary molecular machinery responsible for the docking and the fusion of synaptic vesicles at the chromaffin cell plasma membrane (Figure 2) [174]. Furthermore, it is believed that NSF and SNAPs have multiple sites of actions on SNARE proteins. Besides pre-docking actions and their function as molecular chaperones in SNARE priming, they also act on SNAREs post-fusion to facilitate vesicle retrieval and allow recycling of empty vesicles [145,167,172,174].

Apart from NSF, SNAPs, and SNAREs, several other proteins are involved in calcium triggered exocytosis. Proposed candidates are the stabilizing protein Munc18-1, the calcium transducer calmodulin (CALM), the Ca^2+^ dependent secretion activator (CAPS), rabphilin, and annexins [169,172,174]. Rabphilin3A is a small GTP-ase, which acts as a molecular switch, thereby determining the sensitivity of secretory vesicles for docking and fusion. Overexpression of rabphilin3A has been found to inhibit exocytosis in adrenomedullary chromaffin cells and probably protects against spontaneous exocytosis under basal conditions [175]. Annexins have the ability to form cross-links between secretory vesicles and the plasma membrane by a functional interplay with SNAREs during exocytosis [145,169,176,177,178].

#### 2.3.3. Non-Neuronal Regulation of Catecholamine Secretion

Along with catecholamines, the adrenal medulla synthesizes and releases numerous enzymes, peptides, and proteins that exert various trophic and neurotransmitter activities, which provide fine-tuning of catecholamine synthesis and secretion in an autocrine and a paracrine manner [62,63,64,65,101,111,113,117,118,129,157] (Table 1). We discuss here in more detail neuropeptide Y (NPY), adrenomedullin (AM), PACAP, and the secretion-inhibiting peptide catestatin [101,111,117,118,129]. These peptides modulate chromaffin cell function through a variety of membrane receptors, the vast majority of which belong to the family of G protein-coupled receptors (GPCRs) [117].

Neuropeptide Y is a 36-amino acid neuropeptide, which is widely and abundantly distributed in the brain and the sympathetic nervous system, including the human adrenal medulla. Several NPY receptors (i.e., Y1, Y2, Y4, and Y5) are expressed in the adrenal gland, indicating that NPY exerts local autocrine effects. It has been shown that NPY is able to stimulate catecholamine secretion by inducing TH expression and increasing intracellular calcium [63,101,117,118,129,179].

Adrenomedullin (AM) is a 52-amino acid peptide originally isolated from a human pheochromocytoma and also is present at high concentrations in the normal adrenal medulla [101,117,129]. AM demonstrates autocrine and paracrine effects through binding to the adrenomedullin receptor (ADMR), the receptor dog cDNA (RDC1), and the calcitonin receptor-like receptor (CRLR), which results in augmentation of the adrenal blood flow and stimulation of catecholamine release. In addition, the endocrine effects of AM include systemic vasodilatation and stimulation of natriuresis [63,101,117,129].

As previously mentioned, PACAP not only acts as a neurotransmitter released by the splanchnic nerve but is also co-secreted with catecholamines, exerting its effects by binding to the PACAP-preferring receptor (PAC1-R) and VIP/PACAP receptors (VPAC1-R and VPAC2-R), the former representing the predominant receptor in chromaffin cells (Figure 2) [101,117,129]. PACAP, a neuropeptide of 27 or 38 amino acids, enhances catecholamine secretion by induction of transcription as well as stimulating the activity of the biosynthetic enzymes TH, DBH, and PNMT [101]. In normal adrenal medullary chromaffin cells, PACAP also stimulates the expression and the secretion of several other peptides, such as brain natriuretic peptide, enkephalins, EM66, and secretoneurin, which in turn exert their own individual autocrine/paracrine effects on catecholamine secretion [40,63,101,117,129,130].

Catestatin is a biologically active peptide fragment derived from proteolytic chromogranin A cleavage [63]. Catestatin acts mainly as a noncompetitive nicotinic cholinergic antagonist, thus providing a strong negative feedback inhibition of catecholamine secretion [111,112].

## 3. Pheochromocytoma and Paraganglioma

**Figure 3:**
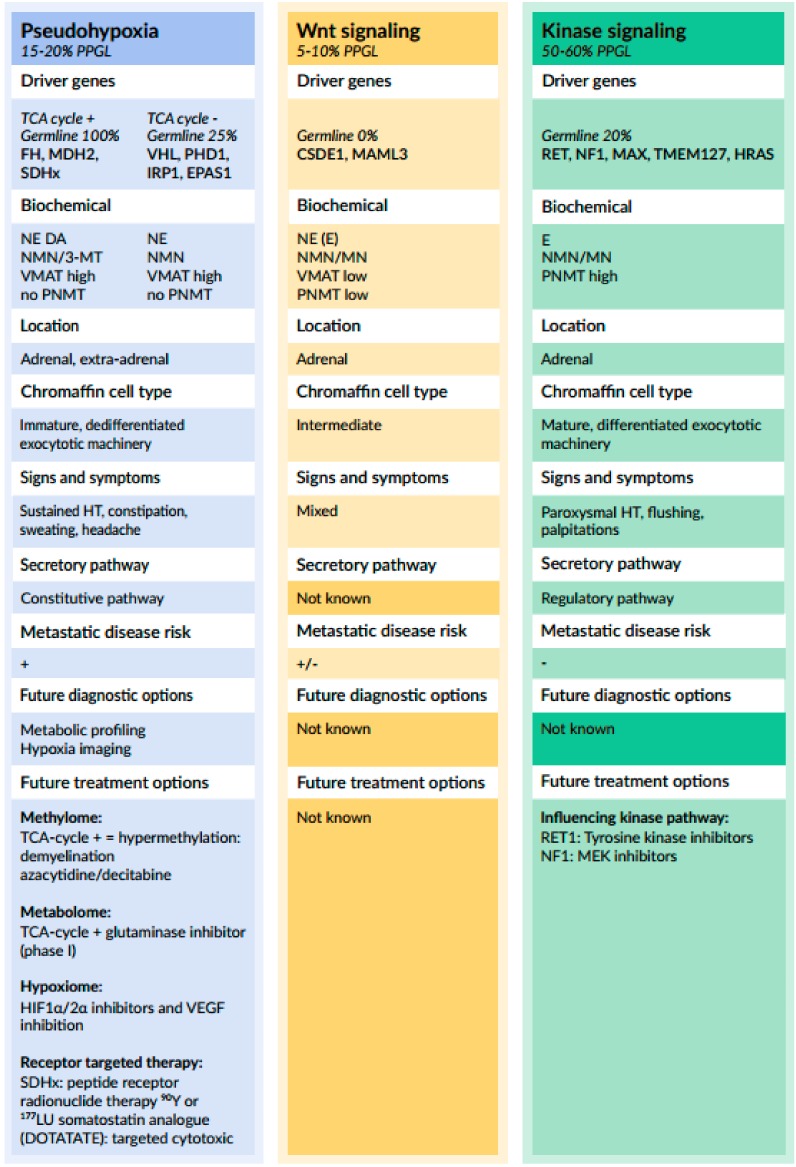
The Pseudohypoxia group (cluster I) divided into two subgroups: tricarboxylic acid (TCA) cycle related, containing germline pathogenic variants in succinate dehydrogenase subunits *SDHA*, *SDHB*, *SDHC*, and *SDHD* as well as *SDHAF2 (SDHx)*, assembly factor for the succinate dehydrogenase complex, and *FH*, a second enzyme in the tricarboxylic acid (TCA) cycle. The second subgroup: *VHL/EPAS1*—related with somatic and germline pathogenic variants. Pathogenic variants in three additional genes encoding for malate dehydrogenase 2 *(MDH2)*, prolyl hydroxylase 1 (*PHD1*, also known as egl nine homolog 2; *EGLN2*), and iron regulatory protein 1 *(IRP1)* were not included previously in the molecular classification by TCGA but were recently discovered. Based on their signaling pathways, it is believed that these new genes should be included as part of the cluster I pseudohypoxia group because *MDH2* is part of to the TCA cycle and both *PDH1* and *IRP1* belong to the *VHL/EPAS1* related subgroup. Cluster I is characterized by the expression of genes involved in the “hypoxic response”, resulting in a “pseudo-hypoxic” phenotype with uncontrolled expression of HIF1α regulated genes such as VEGF. HIF1α regulates the transcription of genes associated with tumorigenesis and angiogenesis. *Wnt altered signaling group (cluster III)* consists of newly recognized somatic mutations in *CSDE1* as well as somatic gene fusions affecting *MAML3*. This group exclusively consists of somatic mutations that activate the Wnt pathway, which is not activated under normal conditions. Wnt signaling and therefore increased expression of β-catenin is associated with a poorer prognosis and a higher metastatic potential of tumors. There is still much unknown about this group. *Kinase signaling group (Cluster II)* consists of germline or somatic pathogenic variants in the driver genes *RET*, *NF1*, *TMEM127*, *MAX*, and *HRAS*. This cluster is characterized by an increased activation of the MAP kinase and the P13K/AKT pathways, which results in an increased expression of genes involved in protein synthesis, kinase signaling, endocytosis, and preservation of differentiated/mature chromaffin cell catecholamine biosynthetic machinery. *MAX* mutated tumors are an exception, since they show an intermediate catecholamine biochemical phenotype with detectable expression of PNMT and some production of epinephrine. *MAX* is a distinct sub-cluster of the kinase signaling group and was recently proposed to be possibly redivided in a new group, the cortical admixture group [187,188,189,193].

From a genetic perspective, pheochromocytomas and paragangliomas (PPGL) have one of the richest hereditary backgrounds among all neoplasms. At least 35—perhaps up to 40%—of all PPGL harbor a germline pathogenic variant in one of the several susceptibility genes [180,181,182,183,184]. Initial gene expression profiling studies by Dahia et al. in 2005 [185] revealed two cluster groups, designated cluster 1 and cluster 2, that reflected respective activation of pseudohypoxia and kinase signaling pathways. Those different gene expression signatures matched closely to those reported in an earlier study for norepinephrine- versus epinephrine-producing sporadic tumors and tumors from patients with von-Hippel Lindau (VHL) syndrome and multiple endocrine neoplasia type 2 (MEN2) [186]. The molecular characterization from The Cancer Genome Atlas (TCGA) project has more recently provided a sophisticated molecular taxonomy of PPGL, which divides these neuroendocrine tumors into groups with similar pathogenesis and molecular biology and provides an up-to-date framework (Figure 3) [187,188,189]. The recent identification of newly recognized somatic mutations in the driver genes, *Cold shock domain-containing E1 (CSDE1)* and *Mastermind-like transcriptional coactivator 3 (MAML3)*, could add a third cluster—i.e., the Wnt altered group [187]—to the original classification of Dahia et al. of 2005 [185]. This cluster 3 is associated with an abnormal activation of the Wnt-signaling pathway.

Cluster 1, the pseudohypoxia group, can be subdivided into a tricarboxylic acid (TCA) cycle- and a *VHL/EPAS1* related group. The TCA cycle-related subgroup consists of germline pathogenic variants in genes encoding fumarate hydratase *(FH)* or one of the succinate dehydrogenase *(SDH)* subunits A, B, C, D, or the complex assembly factor 2 (AF2). Germline pathogenic variants in *Malate Dehydrogenase 2 (MDH2)* and somatic mutations in *Isocitrate Dehydrogenase type 2 (IDH2)* genes also can be categorized in this subgroup [190,191]. The *VHL/EPAS1*-related subgroup consists of germline and somatic pathogenic variants in the genes *VHL* and *EPAS1* [encoding the hypoxia inducible factor 2α (HIF2α) protein] [192]. In addition, it was proposed that mutations in genes encoding prolyl hydroxylase 1 (*PHD1*, also known as egl nine homolog 2; *EGLN2*) and iron regulatory protein 1 *(IRP1)* should also be considered as members of this subgroup [193].

Cluster 2 related mutations are associated with abnormal kinase signaling pathways and include germline or somatic pathogenic variants in genes encoding for rearranged-during-transfection *(RET)*, neurofibromin *(NF1)*, transmembrane protein 127 *(TMEM127)*, MYC-associated factor X *(MAX)*, and Harvey rat sarcoma proto-oncogene *(H-RAS)* [193,194].

Each of these clusters is associated with unique downstream signaling pathways, which correspond to certain clinical features and offer potential targets for future diagnostic, therapeutic, and prognostic purposes (Figure 3).

The hallmark of pheochromocytomas and sympathetic paragangliomas is their ability to secrete catecholamines in an uncontrolled fashion compared to normal adrenomedullary chromaffin cells. Unlike normal adrenomedullary chromaffin cells, chromaffin tumor cells are not innervated, and catecholamine secretion is therefore not stimulated by the previously described neuronal stimuli [195]. Theoretically, and as discussed below, the hypersecretion of catecholamines could be explained by various perturbations of the molecular machinery involved in their biosynthesis, secretion, and metabolism.

### 3.1. Increased Biosynthesis of Catecholamines

The most obvious explanation for the hypersecretion of catecholamines by pheochromocytomas is the increased number of cells able to produce and secrete catecholamines. There are limited data on the tissue concentration of catecholamines in normal adrenal medulla as compared with pheochromocytoma, demonstrating either no difference or a higher content of epinephrine in the latter [88,195]. At the cellular level, there is evidence of an upregulation of expression and activity of the biosynthetic enzymes. In particular, Jarrot et al. [89] described in 1977 that the activity of TH, AADC, and DBH was enhanced in human pheochromocytoma compared to normal human adrenal medulla tissue specimens. These early observations were subsequently supported by several immunohistochemical studies [196,197,198,199,200,201].

Isobe et al. demonstrated that pheochromocytoma cells contained increased levels of mRNA encoding *TH*, *AADC*, and *DBH* compared to normal adrenal medulla [88]. They also found a strong positive correlation between *TH* mRNA concentration and total catecholamine content in pheochromocytomas that was absent in normal adrenal medulla tissue. Moreover, they found lower concentrations of *PNMT* mRNA in pheochromocytoma compared to normal adrenal medulla [88], which might be explained, in part, by lower concentrations of cortisol reaching tumor tissues [88,202]. Of interest, Kimura et al. [196] demonstrated that PNMT immunoreactivity was limited to the mixed epinephrine and norepinephrine producing pheochromocytomas, and tumor cells with PNMT tended to be located close to the adrenal cortex.

All above referenced studies involving comparisons of pheochromocytoma tissue with normal adrenal medulla must be interpreted cautiously since it is difficult to isolate normal adrenal medullary from cortical tissue and thereby establish true differences between normal and tumor cells [203]. What is clear is that catecholamine contents of pheochromocytoma or paraganglioma tumor tissue are highly variable, both in terms of total amounts and relative content of norepinephrine and epinephrine [195,199,204]. Most tumors produce relatively low amounts of dopamine, but there are exceptions, usually isolated paragangliomas presumably lacking significant expression of DBH [205,206,207].

#### 3.1.1. Relationship between Genotype and Catecholamine Biochemical Phenotype

Mutation-dependent differentiation of the chromaffin progenitor cells influences the expression of the biosynthetic enzymes, which leads to distinct phenotypic features of the tumors [206]. Three biochemical phenotypes can be distinguished, i.e., noradrenergic, adrenergic, and dopaminergic, based on the main catecholamines that are produced. Typical pheochromocytomas produce both norepinephrine and epinephrine in variable proportions. A small subset of PPGL is biochemically silent, and almost all of these are paragangliomas [208,209].

The two gene expression clusters (cluster 1 and 2) described earlier are not only characterized by distinct patterns of gene activation but also distinct catecholamine biochemical phenotypes [186,207,210]. Cluster 2 tumors due to somatic and/or germline *NF1*, *RET*, *TMEM127*, *MAX*, and *HRAS* pathogenic variants, which are characterized by activated kinase and protein translation signaling pathways, almost always originate in the adrenals and produce epinephrine due to expression of PNMT. They have more mature catecholamine secretory pathways and phenotypic features [186,207,210] and also tend to develop later in life than tumors due to cluster 1 mutations.

In contrast, cluster 1 tumors with the pseudohypoxic phenotype due to germline pathogenic variants of *VHL*, *SDHx*, *FH*, and somatic mutations of *EPAS1* (HIF2α) occur with variable frequencies at extra-adrenal or adrenal locations and show negligible epinephrine production due to near absent expression of PNMT [186,187,207,210]. This is also independent of their adrenal or extra-adrenal locations due to hypermethylation of the *PNMT* promoter [211]. The underlying pseudohypoxic phenotype of cluster 1 tumors is due to HIF stabilization, and it appears that it is the stabilization of HIF2α that is more important than that of HIF1α for the distinct phenotypic features of cluster 1 compared to cluster 2 chromaffin cell tumors [186,212,213,214,215]. Indeed, HIF2α was identified in 2004 as a key differentially expressed gene, which is more highly expressed in noradrenergic tumors compared to adrenergic tumors, where its expression is essentially absent [186]. Mechanistic studies by Qin et al. [215] showed that expression and the stabilization of HIF2α in chromaffin cells that normally expressed PNMT and produced epinephrine resulted in complete suppression of steroid-induced induction of PNMT. Since HIF2α is expressed transiently in chromaffin progenitors during embryogenesis [216,217,218], it is possible that HIF2α stabilization may promote a noradrenergic rather than an adrenergic phenotype. This concept is supported by findings that transgenic mice with mutations that stabilize HIF2α are characterized by adrenals that express less PNMT and produce more norepinephrine than epinephrine compared to adrenals of wild type mice [219].

Thus, it seems that cluster 1 adrenal tumors arise from chromaffin progenitors in which both transcriptional expression of HIF2α and stabilization of the translated HIF2α protein blocks the effects of steroids produced locally by cortical cells, explaining why these tumors in the end do not produce epinephrine. Such influences occurring during embryogenesis could also be responsible for the younger age of presentation of patients with cluster 1 compared to cluster 2 tumors as well as their propensity for a multifocal presentation [211].

The effect of HIF2α to block steroid induced expression of PNMT and other genes is suggested to involve a mechanism involving the MYC/MAX complex and MYC-mediated control of gene transcription [215]. A role of MAX is indicated by the demonstration that, in rat PC12 pheochromocytoma cells, which lack a functional *MAX* gene, re-expression of MAX facilitates return of steroid-induced PNMT expression. In contrast, silencing MAX in pheochromocytoma cells that express PNMT results in attenuated steroid-induced PNMT [215]. This provides a potential point of intersection for almost all upstream tumor susceptibility genes and also explains the catecholamine biochemical phenotype of MAX-mutated pheochromocytomas, which express some PNMT and produce some epinephrine, though in amounts that lie in between cluster 1 and cluster 2 tumors [207,220].

#### 3.1.2. Relationship between Genotype and Catecholamine Secretory Pathways

The two distinct genetic clusters seem to be not only associated with differences in biochemical profile but also with variations in secretory processes. For example, data derived from microarrays and proteomics have shown reduced expression of various components of the regulated secretory pathway (e.g., SNAP25, syntaxin, rabphilin 3A, annexin) in *VHL*-related pheochromocytomas compared to *RET*-related pheochromocytomas [169]. In addition, the rate constant for baseline catecholamine secretion was found to be 20-fold higher in *VHL*- than in *RET*-related pheochromocytoma. Moreover, only in *RET*-mutated tumors catecholamine, secretion was shown to be responsive to glucagon. These observations suggest that catecholamine secretion in *VHL*-associated pheochromocytomas exhibits more constitutive-like continuous secretory characteristics, whereas in *RET*-associated pheochromocytomas, secretion still is constrained by expression of many components of the regulated secretory pathway. Thus, differences in the molecular machinery underlying catecholamine exocytosis may explain the more paroxysmal nature of symptoms and signs in patients with MEN2 as compared to those with the VHL syndrome. In addition, it was demonstrated that the expression of *VMAT1* mRNA was significantly higher in pheochromocytomas from VHL compared to MEN2 patients [164,189]. In addition, higher levels of *VMAT1* correlated with lower tumor tissue contents of catecholamines and lower numbers of catecholamine containing vesicles, which likely reflects the higher turnover of catecholamines in noradrenergic *VHL*-related compared to adrenergic pheochromocytomas [164].

### 3.2. Alterations in Chromaffin Cell Pathways Associated with Metastatic Pheochromocytoma and Paraganglioma

#### 3.2.1. Clinical Features and Risk Factors

The majority of PPGLs are characterized by a benign clinical course. In 10–15% of cases, however, metastases are present at diagnosis or will develop during follow-up. The most common metastatic sites for chromaffin cell tumors are local lymph nodes, bone, liver, and lung [221]. At the moment, there are no discriminative histopathological features by which the biological behavior of a pheochromocytoma or a paraganglioma can be assessed or predicted reliably [222,223,224,225]. Therefore, the most recent WHO Classification of Endocrine Organs states that all chromaffin cell tumors are considered to have metastatic potential for which long-term follow-up of patients is required, even after successful resection of a pheochromocytoma or a paraganglioma [221].

Several clinical risk factors have been identified that confer an increased risk for development of metastatic disease. Large size (in general > 5 cm) and extra-adrenal location of the primary tumor are associated with metastatic disease [226,227,228,229,230]. Although representing about 20% of the chromaffin cell tumors, sympathetic paragangliomas are the primary source for about 60% of cases with metastatic PPGL [229].

Germline pathogenic variants of the *SDHB* gene are present in a relatively high frequency of 8–10% in patients with a PPGL [184]. Large cohort studies targeted at the genotype–phenotype relationship have shown that, in particular carriers of an *SDHB* germline, pathogenic variants have an increased risk of metastatic PPGL [184,230,231,232]. Typical *SDHB* related PPGLs occur at a younger age and arise more frequently in extra-adrenal locations [233]. A possible increased predisposition to metastatic disease has been suggested for rare germline pathogenic variants in *FH* [234], *SLC25A11* [235], *SDHA*, and *TMEM127* [236], although the number of cases is quite low, making the association difficult to confirm.

In addition to size and location of the PPGL and the presence of certain germline mutations, the risk of metastatic disease is also increased in association with certain alterations in the biochemical profile [237]. In particular, plasma concentrations of dopamine and its metabolite 3-methoxytyramine are significantly higher in patients with metastatic PPGL compared to those with non-metastatic disease [237,238]. Most patients with metastatic PPGLs demonstrate a noradrenergic profile with elevated plasma levels of both norepinephrine and normetanephrine, with concomitant lack of or negligible relative increases in plasma concentrations of epinephrine and metanephrine compared to subjects without metastatic disease [237,239]. In a relatively large series of patients with metastatic PPGL, only a minority (11%) of patients had an adrenergic phenotype. Of note, none of these patients had a *SDHB*-related pheochromocytoma or paraganglioma [240]. The biochemical profile has also been linked to prognosis, as it was shown that patients with elevated plasma levels of dopamine and norepinephrine had a faster progression of their disease [239].

#### 3.2.2. Molecular Alterations in SDHx-Related PPGL and Their Effect on Catecholamine Biosynthesis

The SDH complex is a hetero-tetrameric mitochondrial enzyme that consists of two catalytic subunits (SDHA and SDHB) and two membrane-anchoring subunits (SDHC and SDHD). SDH catalyzes the oxidation of succinate to fumarate in the TCA cycle and transfers electrons to the ubiquinone (coenzyme Q) pool in the respiratory chain. SDH assembly factor (SDHAF) is required for the flavination of SDHA, an essential step in formation of the SDH complex.

SDH deficiency leads to the accumulation of succinate, which has structural similarity to 2-ketoglutarate. Succinate is therefore able to act as a competitive inhibitor of 2-ketogluarate-dependent dioxygenases, which include prolyl hydroxylase domain proteins (PHDs), ten-eleven translocation (TET) enzymes, and jumonji-domain histone demethylases (JmjC) demethylases [241,242]. This results in hypermethylation of CpG (cytosine preceding guanine) islands, regions within the genome that are common in promoter sites rich in CpG dinucleotides. Succinate is a typical example of an oncometabolite, a metabolite that abnormally accumulates in cancer cells as a result of a defective gene encoding the corresponding enzyme, thereby modifying signaling pathways and epigenetic regulation mechanisms. In the study by Letouzé et al. [211], the level of hypermethylation was significantly higher in *SDHB* compared to other *SDHx* mutated tumors. Of interest, it was shown that succinate:fumarate ratios were higher in tumor tissue derived from patients with a *SDHB* pathogenic variant as compared from those with a *SDHC/D* pathogenic variant [243]. This suggests that functional activity of the SDH complex is most disrupted in the case of mutations of the SDHB subunit, resulting in higher intracellular concentrations of oncometabolites and a concurrent higher metastatic risk.

PHDs are involved in the inactivation of the HIF, a heterodimer that consists of two subunits, one α subunit and one β subunit. There are two different α-subunits (HIF1α and HIF2α) and two different β subunits (HIF1 β and aryl hydrocarbon receptor nuclear translocator ARNT2). The β subunits are constitutively expressed, whereas HIF1α and HIF2α are inactivated in the presence of oxygen through hydroxylation by PHDs and subsequent degradation by the VHL–ubiquitination complex (pVHL). The hydroxylation reaction performed by the PHDs requires oxygen and α-ketoglutarate as substrates as well as iron and ascorbate as cofactors [244].

Thus, PHD is inactive in the presence of hypoxia, resulting in stabilization of HIFα. The unmodified HIFα molecule translocates to the nucleus, where it forms a transcriptionally active heterodimer together with a HIFβ subunit, which is able to stimulate various target genes involved in angiogenesis, energy metabolism, and cell survival.

The epigenetic modifications are thought to play an important role in tumorigenesis by deregulating gene expression of key genes. Methylome analysis of a large PPGL cohort demonstrated a clear hypermethylator phenotype in the *SDHx*-related tumors [211].

Besides the *PNMT* gene, three other genes involved in the catecholamine pathway were also found to be hypermethylated, i.e., *DRD2*, *NPY*, and *SLC6A2* [211]. Transcription of *DRD2* results in the synthesis of the D2-dopamine receptor, and the *SLC6A2* gene or *solute carrier family 6, member 2* gene encodes the norepinephrine transporter (NET) responsible for reuptake of norepinephrine into presynaptic nerve terminals.

#### 3.2.3. Relationship between Other Components of the Exocytotic Machinery and Metastatic Disease

The co-secreted neuropeptides neuropeptide Y, adrenomedullin, and PACAP are overexpressed in pheochromocytomas and stimulate catecholamine release [129]. These neuropeptides have also been implicated in influencing cell survival and tumor growth of PPGLs (Table 1) [119,120,129]. High expression levels of the adrenomedullin receptor RDC1 were demonstrated in a small series of metastatic PPGL. Overexpression of the adrenomedullin receptor RDC1 has been described in several cancers and has been found to be associated with invasiveness, survival, proliferation, and neo-angiogenesis [129]. These observations suggest a pathophysiological role of the adrenomedullin receptor RDC1 in metastatic PPGL.

Recently, overexpression of LAT-1, and to a lesser extent of LAT-2, has been demonstrated in pheochromocytoma. Moreover, LAT-1 overexpression was strongly correlated with higher levels of urinary catecholamine excretion. It seems plausible that this enhanced expression of LAT is required to ensure a sufficient supply of tyrosine as substrate for the increased catecholamine synthesis. Of interest, LAT-1 expression has been described as a poor prognostic marker in various malignancies, including lung, pancreas, breast, and hepatocellular cancer [245]. It is currently unknown whether LAT expression has any prognostic value in PPGL [246,247,248,249].

Connexins (Cx) are the specialized proteins of gap junctions, and these structures play an important role in cell proliferation and differentiation as well as in carcinogenesis. The connexion family consists of 21 different proteins, and it has been demonstrated that the expression pattern of connexins in pheochromocytomas is different from normal adrenal medulla tissue [170,250]. In addition, the expression of Cx50 was lower in metastatic as compared to benign pheochromocytomas [250]. However, data on the association between connexion expression pattern and biological behavior of pheochromocytomas are very limited.

In summary, our understanding of the intracellular molecular intricacies associated with metastatic PPGL has greatly improved in recent years. Although elucidation of these pathways is still incomplete, research of the genome–metabolome–phenotype relationship has already generated exciting and clinically important information of these rare neuro-endocrine tumors.

## 4. Conclusions and Future Perspectives

In conclusion, our increased knowledge of catecholamine synthesis, secretion, and regulation has improved our understanding of chromaffin cell tumorigenesis. Disregulation of these pathways is evident in pheochromocytomas and paragangliomas and varies between the different genomic backgrounds of the tumors. For example, the differences in PNMT, VMAT, RDC1, and LAT1 expression may signify a difference in cellular dedifferentiation, making certain tumors more aggressive. In addition, substances such as neuropeptide Y, PACAP, EM66, bombesin, and connexins might be differentially expressed by benign and metatstatic PPGL. These potential prognostic biomarkers will need to be examined in larger and more broad cohorts of PPGLs and in prospective studies to determine their true potential utility as prognostic markers. Moreover, further studies of chromaffin cell products with an unknown role in PPGL (Table 1) might also reveal clinically useful information.

Future research will continue to provide novel and relevant information that will enhance our current knowledge with respect to both the physiology and the pathophysiology of the chromaffin cell. To this end, an integrative and translational approach is required by combining clinical information with (epi)genomics, transcriptomics, and metabolomics. Such new information could, for example, elucidate the precise relationship between the net effect of the mixture of substances that are co-secreted with catecholamines and the clinical picture. In addition, it would allow better discrimination between benign and potentially metastatic PPGL at the time of initial diagnosis. Moreover, improved insight into the molecular pathways that drive the transformation of the normal chromaffin cell into the malignant chromaffin cell will also offer novel targets for treatment that hopefully will provide a definitive cure for these rare metastatic neuroendocrine tumors in the future.

## Figures and Tables

**Table 1:** Overview of the co-secreted products of chromaffin vesicles with description of their function in normal adrenal medulla and reported alterations in pheochromocytoma and paraganglioma (PPGL).

Component	Function in Human Adrenal Medulla	Reported Alterations in PPGL
**Granins**		
Chromogranin A–C [65,66,67,68,69,70,71,72,73,74,75,76,77]	Role in vesiculogenesis, vesicle protein stability, hormone storage within vesicles. Sorting proteins in the regulated secretory pathway. Precursor protein for several peptides; chromogranin A (vasostatin I–II, catestatin, cateslytin, chromacin, chromofungin, pancreastatin, parastatin, WE-14, EL35), chromogranin B (secretolytin), chromogranin C (secretoneurin, EM66, manserin).	Higher plasma levels of chromogranin A and B are reported in PPGL compared to healthy volunteers. Chromogranin C mRNA was overexpressed in PPGL compared to non-tumoral chromaffin tissue. Higher expression of chromogranin A and B in *RET* associated PPGL compared to *VHL* associated PPGL at both the mRNA and protein levels. Downregulation of chromogranin B and chromogranin C observed to be associated with malignant behavior.
Secretogranins III–VII [78,79,80,81,82,83,84,85,86,87]	Secretogranin III (syn. 1B1075) not found in adrenal medulla. Presence of secretogranin IV (syn. HISL-19), V (syn. 7B2), VI (syn. NESP55) and VII (VGF) reported in human adrenal medulla, exact function still mainly unknown. Proposed role of secretogranin VII (syn. VGF) in the regulation of energy homeostasis.	More pronounced immunoreactivity of secretogranin IV in malignant PPGL compared to benign PPGL. Significantly higher plasma levels of secretogranin V in PPGL compared to age-matched normal subjects. Secretogranin VI immunoreactivity found in PPGL with no differences between benign and malignant tumors. Variable proVGF-immunoreactive fragments are observed in human PPGL.
**Glycoproteins**		
Glycoprotein I–V [88,89,90]	Glycoprotein I, i.e., DBH, catalyzes the conversion of dopamine into norepinephrine. Glycoprotein IV, i.e., the H^+^-ATP-ase subunit M45, provides the driving force for vesicular uptake of catecholamines by VMAT.	High expression levels of glycoprotein I (DBH) are reported in PPGL.
**Prohormone processing enzymes**		
Aminopeptidase B (Ap-B) [91]	Exopeptidase involved in final conversion of proenkephalin to enkephalin.	Association of Ap-B with the secretory machinery is suggested in rat pheochromocytoma (PC12) cells.
Aspartic Proteinase [92]	Contributes to enkephalin precursor cleaving activity.	Unknown
Carboxypeptidase E (CPE) [93,94]	Role in peptide processing and sorting of prohormones.	High expression of CPE mRNA are reported. Elevated expression correlated with tumor growth and metastasis in pheochromocytomas. CPE promotes survival of pheochromocytoma (PC12) cells under nutrient starvation and hypoxic conditions by upregulation of pro-survival genes possibly by activation of the ERK1/2 pathway.
Cathepsin L [95,96]	Endopeptidase involved in proteolysis of proenkephalin into (Met)enkephalin. Proprotein convertase for biosynthesis of NPY and catestatin.	Unknown
Prohormone convertase 1/3 and 2 [73,78,97]	Conversion of chromogranin C into secretoneurin and EM66.	*PC1* and *PC2* mRNA expression levels are significantly higher in benign and malignant PPGL compared to normal adrenal medulla. mRNA expression and protein levels of *PC1* and *PC2* is 3–4 times higher in benign tumors compared to malignant tumors.
Tissue-Type Plasminogen Activator (t-PA) [98,99]	Participation in plasmin-dependent processing of bioactive peptides including chromogranin A and indirectly modulate chromogranin A release (negative-feedback loop).	Marked expressions of t-PA mRNA are reported in human pheochromocytomas.
**Inhibitors of endogenous proteases**		
Endopin 1–2 [100]	Endopin 1 inhibits trypsin-like serine proteases. Endopin 2 inhibits papain-like cysteine proteases, including cathepsin L, as well as the serine protease elastase.	Unknown
**Transmitter peptides**		
Adrenomedullin [65,101,102,103,104,105]	Increases blood flow in the adrenal gland. Increases catecholamine release. Induces systemic vasodilation. Increases natriuresis.	High plasma levels are reported, especially in PPGL patients with high blood pressure.
Bombesin [106,107,108]	Modulation of stress response. Paracrine regulatory effects on growth, structure and function of the adrenal cortex.	Highly variable immunoreactivity in pheochromocytomas and paragangliomas. An association between clinically malignant PPGL and lower expression of bombesin is postulated.
Calcitonin Gene-Related Peptide [109,110]	Vasodilatation, enhances aldosterone and corticosterone release by adrenocortical cells.	Slightly elevated levels are reported.
Catestatin, Cateslytin [111,112]	Inhibits release of catecholamines, chromogranin A, NPY, and ATP by acting as noncompetitive antagonist of the nicotinic receptor.	Unknown
EM66 (via PACAP) [73,113,114]	Derivate from chromogranin C (syn. secretogranin II). Synthesis and secretion regulated by PACAP. Paracrine regulation of steroidogenic cells in the adrenal gland.	Elevated in PPGL. Higher levels of EM66 reported in benign vs. malignant PPGL.
Natriuretic peptides (ANP, BNP, CNP) [102,115,116]	Autocrine/paracrine inhibition of catecholamine secretion via the ANF-R2 receptor subtype. Inhibition of aldosterone production by a direct action on the adrenal cortex both in vivo and in vitro.	Elevated in PPGL patients with high blood pressure.
Neuropeptide Y (NPY) [65,76,101,117,118,119,120,121,122]	Increases catecholamine biosynthesis (stimulates *TH* gene expression) and secretion. Potentiates catecholamine induced vasoconstriction.	Influence on tumorigenesis and stimulation of neoangiogenesis is described. High levels of NPY mRNA have been found in benign tumors, whereas its plasma levels are elevated in patients with malignant PPGL. Significantly lower expression level of NPY in *VHL* associated PPGL compared to other hereditary and sporadic PPGL is reported.
Neurotensin [123,124]	Not detected in human adrenal medulla. Various central and peripheral effects have been postulated in bovine, cat and rat, e.g., hypotension, hypothermia, analgesia.	Neurotensin has rarely been demonstrated in human PPGL.
Opioid peptides (enkephalins, endorphins) [96,117,125,126,127,128]	Decrease catecholamine release via binding G_i_ protein coupled receptor resulting in inhibition of Ca^2+^ channels Analgesia, enhancement of immune reaction.	Enkephalin decreases norepinephrine release in human pheochromocytomas. Different enkephalins (i.e., (Met)enkephalin, (Leu)enkephalin) are observed in human pheochromocytomas. Expression of (Met) enkephalin and (Leu)enkephalin is highly variable compared to normal adrenal medulla. Possible association between malignant PPGL and lower expression of enkephalins.
PACAP [40,63,101,117,129,130]	Induces transcription and stimulates activity of TH, DBH, and PNMT Stimulates expression and secretion of several other peptides e.g., brain natriuretic peptide, enkephalins, EM66, and secretoneurin.	High mRNA expression of PACAP and PAC1-R in PPGL reported. Comparable expression levels in benign and metastatic PPGL found in a relatively small series.
Secretoneurin (via PACAP) [131,132,133]	Derivate from chromogranin C (syn. secretogranin II). Synthesis and secretion regulated by PACAP. Role in angiogenesis and modulation of inflammatory response by chemoattractant effects on monocytes, eosinophils, fibroblasts, vascular smooth muscle cells, and endothelial cells.	Unknown
Transforming Growth Factor β [134,135,136]	Role in regulation of chromaffin cell proliferation and differentiation. Reduction of TGF β has been shown to increase proliferation of chromaffin cell in vivo.	Unknown
Vasostatins [137,138,139]	The N-terminal fragment of chromogranin A Inhibition of endothelin-induced vasoconstriction Antibacterial and antifungal activity.	Unknown
**Anti-bacterial/anti-fungal peptides**		
Chromacin P, G and PG [140]	Antibacterial activity against Gram positive bacteria.	Unknown
Secretolytin [141]	Antibacterial activity against Gram positive bacteria.	Unknown
Ubifungin [142]	Antifungal activity.	Unknown
Other minor components		
Ascorbic acid [143]	Regulation of DBH activity.	Unknown
Coenzyme A glutathione disulfide [144]	Vasoconstriction, modulation of AngII effects.	Unknown
Ions (Ca^2+^, Na^+^, K^+^, Mg^2+^, Cl^−^) [145]	Regulation of exocytosis.	Unknown
Galanin [81,146,147]	Stimulation of norepinephrine and glucocorticoid secretion.	Immunoreactivity in human PPGL. Higher levels reported in PPGL compared to normal adrenal medulla. Variable expression; galanin was predominantly found in noradrenergic pheochromocytoma cells. Induction of apoptosis in PC12 cells. Inhibition of dopamine secretion in pheochromocytoma cells.
Nucleotides (ATP, ADP, GTP) [148]	Formation of intravesicular complex with catecholamines, buffer function, decreases intravesicular osmotic pressure, neuromodulation.	Unknown
Substance P [149,150,151,152,153]	Inhibition of nicotinic acetylcholine receptor mediated catecholamine release. Vasodilation.	Variable immunoreactivity demonstrated in human pheochromocytomas. Elevated plasma levels in minority of patients.
Vasoactive intestinal polypeptide [154,155,156]	Stimulation of catecholamine release, stimulation of steroid secretion.	Few cases described of human PPGL with concomitant excessive VIP secretion.

Abbreviations: ANF-R2, atrial natriuretic factor receptor subtype 2; AngII, angiotensin II; CGA, Chromogranin A; CGB, Chromogranin B; CGC, Chromogranin C; DBH, dopamine β-hydroxylase; HISL-19, human islet cell antigen 19; NESP55, neuroendocrine secretory protein 55; NPY, Neuropeptide Y; PACAP, pituitary adenylate cyclase-activating polypeptide; PAC1-R, PACAP-preferring receptor; PC, Prohormone-Converting Enzymes; TH, tyrosine hydroxylase.
